# The Current Evidence of Intensity-Modulated Radiotherapy for Hepatocellular Carcinoma: A Systematic Review and Meta-Analysis

**DOI:** 10.3390/cancers15204914

**Published:** 2023-10-10

**Authors:** Won Il Jang, Sunmi Jo, Ji Eun Moon, Sun Hyun Bae, Hee Chul Park

**Affiliations:** 1 Department of Radiation Oncology, Korea Institute of Radiological and Medical Sciences, 75, Nowon-ro, Nowon-gu, Seoul 01812, Republic of Korea; zzang11@kirams.re.kr; 2 Department of Radiation Oncology, Haeundae Paik Hospital, Inje University School of Medicine, 875, Haeun-daero, Haeundae-gu, Busan 48108, Republic of Korea; astraea00@naver.com; 3 Department of Biostatistics, Soonchunhyang University College of Medicine, Bucheon, 170 Jomaru-ro, Wongmi-gu, Bucheon-si 14584, Gyeonggi-do, Republic of Korea; moon6188@schmc.ac.kr; 4 Department of Radiation Oncology, Soonchunhyang University College of Medicine, Bucheon, 170 Jomaru-ro, Wongmi-gu, Bucheon-si 14584, Gyeonggi-do, Republic of Korea; 5 Department of Radiation Oncology, Samsung Medical Center, Sungkyunkwan University School of Medicine, 81 Irwon-ro, Gangnam-gu, Seoul 06351, Republic of Korea

**Keywords:** hepatocellular carcinoma, intensity-modulated radiotherapy, meta-analysis, radiotherapy

## Abstract

**Simple Summary:**

Intensity-modulated radiotherapy (IMRT) is used worldwide for all tumor sites, for both curing and palliation. While evidence exists for lower normal tissue toxicity, IMRT did not show a definitive survival benefit compared to non-modulated RT. This is the first systematic review and meta-analysis to evaluate the rationale of IMRT for HCC in the liver. Although most patients had advanced-stage HCC and combined treatment were commonly used, IMRT for HCC showed similar survival with existing RT modalities and relatively low severe toxicity.

**Abstract:**

Intensity-modulated radiotherapy (IMRT), an advanced RT technique, is a considerable treatment option for hepatocellular carcinoma (HCC). However, the distinguishing features of IMRT for HCC have not yet been clearly defined. A systematic review was performed according to the guidelines of the Preferred Reporting Items for Systematic Reviews and Meta-Analyses. The PubMed/MedLine, Embase, Cochrane Library, Web of Science, and KoreaMed were used to screen eligible studies focusing on treatment outcomes after IMRT for HCC until 18 April 2023. A total of 1755 HCC patients receiving IMRT among 29 studies from 2009 to 2023 were selected for the meta-analysis. The median proportion of Barcelona Clinic Liver Cancer stage C was 100% (range: 38–100%). Nineteen studies used combined treatment. Pooled rates of response and 1-year local control were 58% (95% confidence interval [CI], 50–65%) and 84% (95% CI, 70–94%), respectively. The median overall survival (OS) was 13 months (range: 5–45 months), and pooled 1- and 3-year OS rates were 59% (95% CI, 52–66%), and 23% (95% CI, 14–33%), respectively. Pooled rates of classic radiation-induced liver disease (RILD), nonclassic RILD, and hepatic toxicity ≥ grade 3 were 2%, 4%, and 4%, respectively. Although most patients had advanced-stage HCC and combined treatment was commonly used, IMRT for HCC showed similar survival to existing RT modalities and relatively low severe toxicity.

## 1. Introduction

Hepatocellular carcinoma (HCC) is the most common primary liver cancer and the leading cause of cancer-related deaths worldwide [1]. A wide range of treatment options are available for HCC, including hepatic resection, liver transplantation, locoregional ablative therapies, transarterial chemo-embolization (TACE), external-beam radiation therapy (EBRT), and systemic therapies [2]. Historically, EBRT for HCC has been used restrictively because of the relative radiosensitivity of the normal liver and technological limitations in tumor delineation and RT delivery [3]. With the application of three-dimensional conformal radiotherapy (3DCRT), intensity-modulated radiotherapy (IMRT), stereotactic body radiotherapy (SBRT), and charged-particle therapy (CPT), however, RT for HCC has undergone a series of technical advances over the decades, and the indications of RT have been expended to cure [4,5,6].

IMRT was introduced in the early 1990s as a further refinement of the delivery of 3DCRT. This advanced RT technique is based on the use of optimized nonuniform radiation beam intensities with an inverse planning treatment system and can deliver a higher dose to the tumor while sparing normal organs [7]. IMRT is used worldwide as a standard tool for all tumor sites and has been systemically reviewed to support the rational use of IMRT for head and neck cancer, breast cancer, prostate cancer, lung cancer, and so on [8,9]. However, in case of HCC, it remains unclear whether IMRT can have clinically relevant advantages. One meta-analysis compared treatment outcomes for HCC according to RT modality [10]. The authors reported that CPT resulted in significantly higher survivals and lower toxicity than conventional RT, including 3DCRT and IMRT, whereas comparable efficacy and acute toxicity were observed between CPT and SBRT. Among the 10 studies included in the conventional RT group, however, only 1 study treated HCC patients with IMRT (14%) [11]. The conventional RT group mainly included patients with palliative intent who underwent RT for macrovascular invasion (MVI) or similar diseases [12].

Therefore, we conducted a systematic review and meta-analyses to investigate the evidence for the use, and to define distinguishing features, of IMRT for HCC.

## 2. Materials and Methods

This systematic review was conducted according to the Preferred Reporting Items for Systematic Reviews and Meta-analyses guideline [13]. Registration of systemic review is recommended for the following reasons: 1. to prospectively register a systemic review, and 2. to provide an oversight of all registered systemic reviews and reduce the unintended duplication of systemic reviews [14]. This systemic review was conducted on schedule without its registration in PROSPERO because there is no information on the overall processing time in PROSPERO and some have reported that it may take up to several months [15]. Consequently, the whole process from the data search to submission had been performed within 4 months. We published the review journal about IMRT for HCC in 2017 and firstly planed a systemic review using accumulated clinical studies [16].

A literature search was performed using the PubMed/MedLine, Embase, Cochrane Library, Web of Science, and KoreaMed databases. The keywords used in the patient/problem, intervention, comparison, and outcome (PICO) model are provided in the Supplementary Materials. The search strategy was developed and reviewed by the authors in collaboration with a professional librarian at Soonchunhyang University College of Medicine, Bucheon. Full-text articles on humans published in English between 1998 and April 2023 were identified to include modern IMRT techniques [7,17]. A total of 950 articles were identified and 2 authors (W.I. Jang and S. Jo) independently screened the article titles, abstracts, and full texts as necessary. Disagreements were resolved by a third author (S.H. Bae).

Studies which met the following inclusion criteria were considered for the meta-analysis: (1) prospective or retrospective studies to treat the tumors in the liver with IMRT; (2) the description of IMRT techniques using the inverse treatment planning process including step-and-shoot IMRT, sliding-window IMRT, volumetric modulated arc therapy, or helical tomotherapy for the liver lesion; (3) IMRT performed in ≥10 fractions; (4) inclusion of ≥10 HCC patients; and (5) reporting of at least local control (LC) and/or survivals. In the absence of numeric data, LC and survivals were indirectly assumed using descriptive plots. In treatment centers with multiple publications, the largest publication was selected. However, studies from a single center were independently categorized if they were reported in distinct period. In addition, the 2 treatment groups in one study were independently categorized if LC and survivals were reported separately. Studies were excluded if IMRT was (1) applied for neoadjuvant or adjuvant treatment for surgery; (2) originally designed as a bridge to liver transplantation; (3) conducted by Cybernife, Gammaknife, brachytherapy, or CPT; and (4) treated only for distant metastatic lesions.

Data extraction was performed to obtain the following: (1) basic information of the patients and tumor; (2) details of intervention measures; (3) treatment outcomes; and (4) hepatic and gastrointestinal (GI) toxicity rates. The best overall treatment response was evaluated using the Response Evaluation Criteria in Solid Tumors (RECIST), the modified RECIST (mRECIST), or the World Health Organization criteria. The objective response rate (ORR) was defined as the proportion of patients with a complete response (CR) and partial response (PR). Disease control rate (DCR) was defined as the sum rate of CR + PR + stable disease. Survival rates at 1 to 3 years were evaluated. Hepatic toxicity was assessed in accordance with the Common Terminology Criteria for Adverse Events (CTCAE) or the Radiation Therapy Oncology Group (RTOG), and/or radiation-induced liver disease (RILD), of which there are 2 types: classic RILD and nonclassic RILD, which was a little differently defined among included studies. Data on patients with severe gastrointestinal ulcer, bleeding, and/or perforation ≥ grade 3 were collected.

Because most studies were retrospective, the included study quality was assessed using the Newcastle–Ottawa Scale (NOS) [18]. Studies over 7 points were categorized as high-quality, and studies with a score of 4–6 were categorized as medium-quality.

The Higgins I^2^ statistic was used to assess heterogeneity in this meta-analysis [19]. An I^2^ value of ≥50% was thought to represent substantial heterogeneity. Because of the variability in IMRT indication, different study periods among studies, and different etiology according to country, the random-effects model derived from the DerSimonian and Laird method was adopted [20]. Funnel plots were used to evaluate publication bias and the symmetry of the funnel plots was quantitatively analyzed using Egger’s regression tests. If the funnel plot was symmetrical or the *p* value was >0.05 in Egger’s test, then the null hypothesis of no publication bias was accepted. For comparison between subgroups, a Q test based on the analysis of variance and a random-effects model was used. Statistical significance was set at *p* < 0.05. Rex Excel-based statistical analysis software, ver. 3.6.0 (RexSoft, Seoul, Republic of Korea; http://rexsoft.org, accessed on 23 June 2023) was used for statistical analyses.

## 3. Results

### 3.1. Studies’ Characteristics

**Figure 1:**
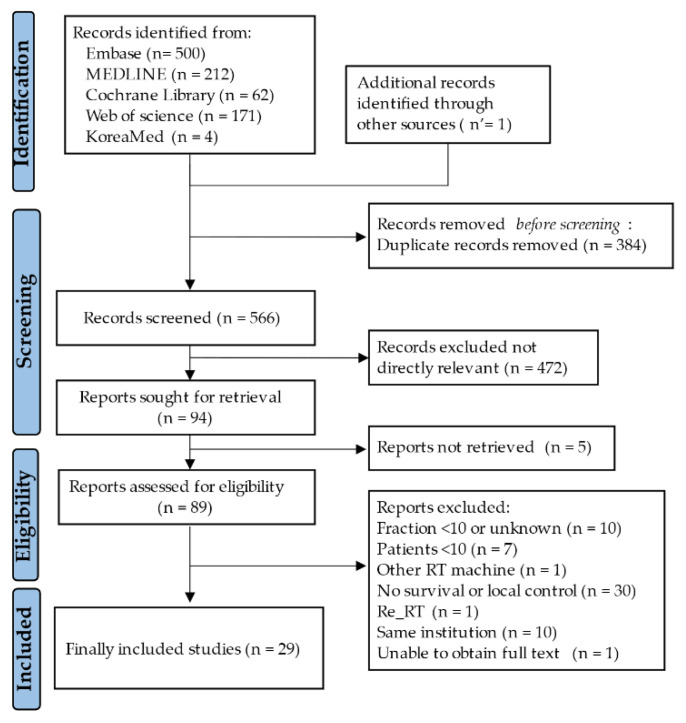
The process of study selection.

Among the 950 initially included studies, 384 were excluded due to duplications between databases. The remaining 566 studies were screened by their title and abstract review, of which a full-text review was conducted in 89 studies. Finally, 1755 patients from 29 studies were eligible for this systematic review and meta-analysis, as shown in Figure 1 [21,22,23,24,25,26,27,28,29,30,31,32,33,34,35,36,37,38,39,40,41,42,43,44,45,46,47,48,49].

Among these, 4 studies separately reported the outcomes of patients treated with IMRT in two treatment groups, and each treatment group was categorized into a different cohort [29,30,32,37]. Therefore, a total of 33 cohorts were included in this study.

The characteristics of the 33 cohorts in the 29 studies are summarized in Table 1 and Table 2. A total of 6 were prospective studies, and 23 were retrospective studies. The quality of each study according to NOS is presented in Table 1.

All the studies, except one, were conducted in Asia. Patients with Child–Pugh (CP) class A was 39–100% (median: 79%). Most patients had advanced HCC, and the median proportion of Barcelona Clinic Liver Cancer (BCLC) stage C was 100% (range: 38–100%). The tumor size ranged from 2.3 cm to 11.4 cm (median: 7.4) and 0–80% of the patients (median: 38%) had multiple HCCs in the liver. MVI and distant metastases were presented in 0–100% (median: 100%) and 0–100% (median: 0%). The median proportion of patients with alpha-fetoprotein (AFP) levels above 400 ng/mL before IMRT was 43% (range: 24–67%). The median total IMRT dose was 51 Gy (range: 42–62 Gy). Nineteen studies used a combined treatment with TACE, systemic chemotherapy, targeted agents, and/or immunotherapy.

### 3.2. Response and Survivals

**Figure 2:**
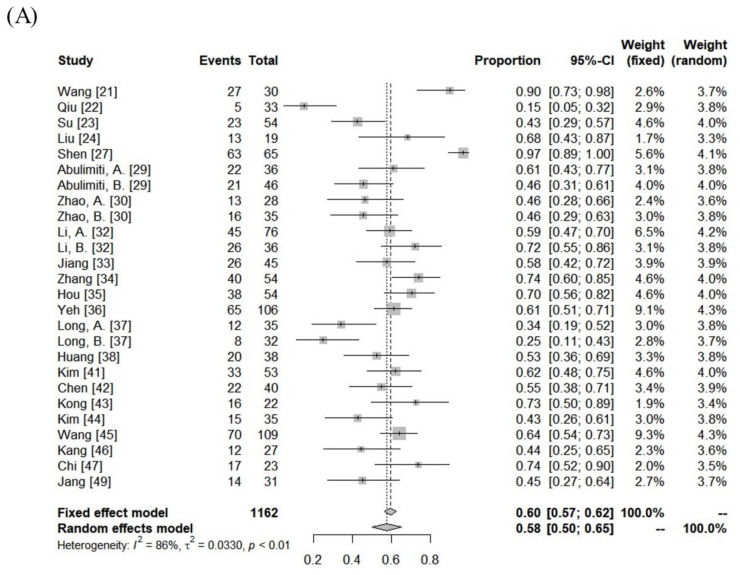

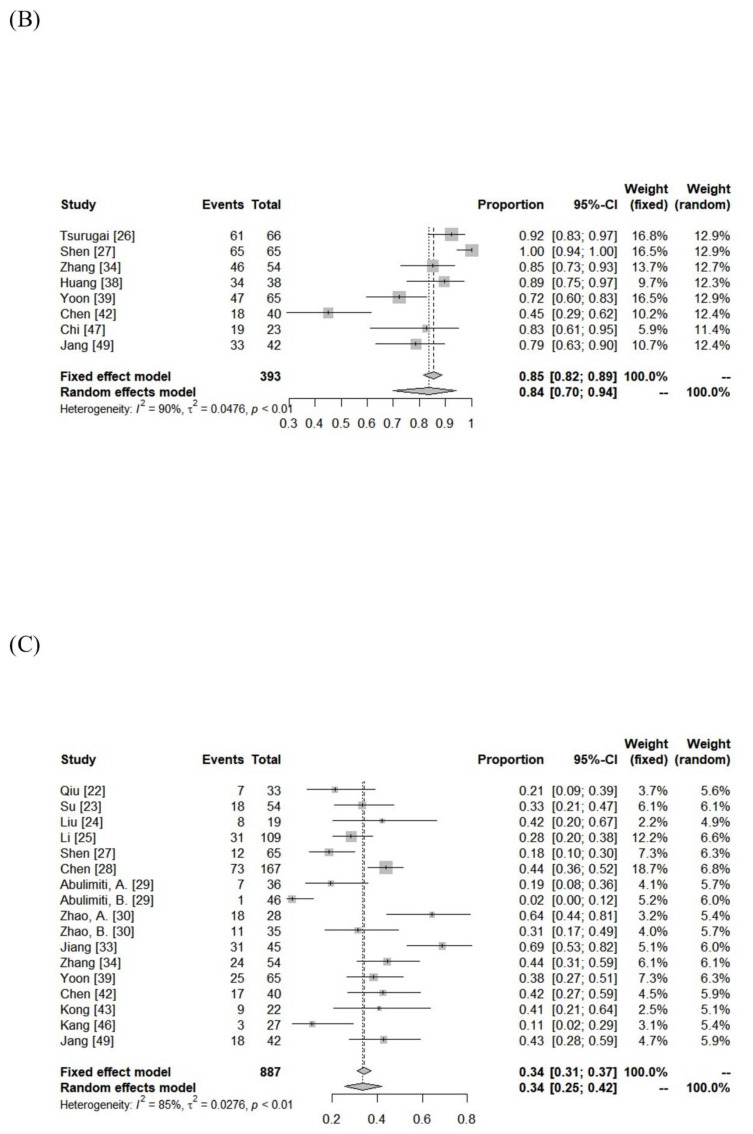

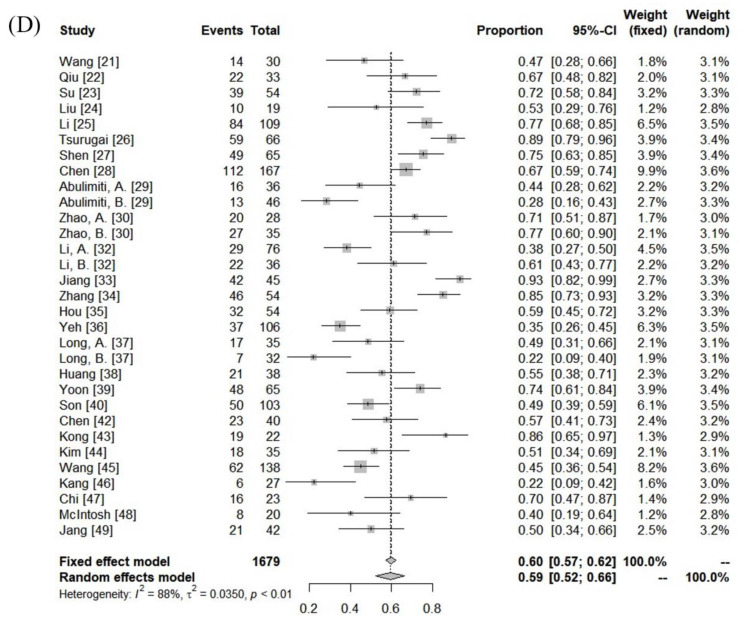
Forest plot of objective response rate (**A**) and local control rate (**B**), progression-free survival rate (**C**), and overall survival rate (**D**) at 1 year.

The median follow-up period was 13 months (range: 5–52 months). The ORR and DCR were 15–97% (median: 62%) and 74–97% (median: 92%). Using random-effects analysis, the pooled ORR and DCR were 58% (95% confidence interval [CI], 50–65%), and 90% (95% CI, 87–93%), respectively. The median 1- and 3-year LC rates were 83% (range: 45–100%) and 67% (range: 47–77%). The median 1- and 3-year progression-free survival (PFS) rates were 39% (range: 3–69%) and 13% (range:0–24%). The median overall survival (OS) was 13 months (range: 5–45 months), and the 1- and 3-year OS rates were 57% (range: 22–93%) and 22% (range: 0–69%). The pooled 1- and 3-year LC rates were 84% (95% CI, 70–94%) and 64% (95% CI, 45–81%), respectively. The pooled PFS and OS rates were 34% (95% CI, 25–42%) and 59% (95% CI, 52–66%) at 1 year and 9% (95% CI, 2–20%) and 23% (95% CI, 14–33%) at 3 years, respectively (Figure 2).

There was significant heterogeneity between the cohorts (Table 3), but no publication bias was detected, except for DCR (Supplementary Figure S1). In the subgroup comparison, a median tumor size ≤7 cm was the only statistically favorable parameter for 1-year OS (*p* < 0.05), as summarized in Table 3.

### 3.3. Toxicities

**Figure 3:**
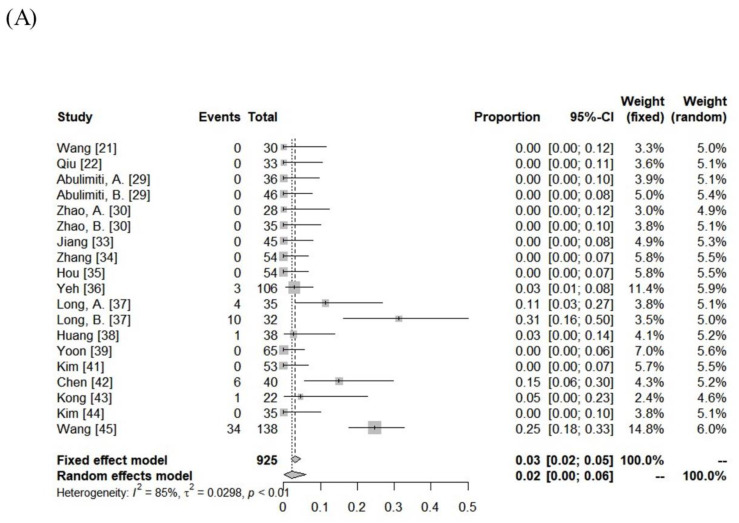

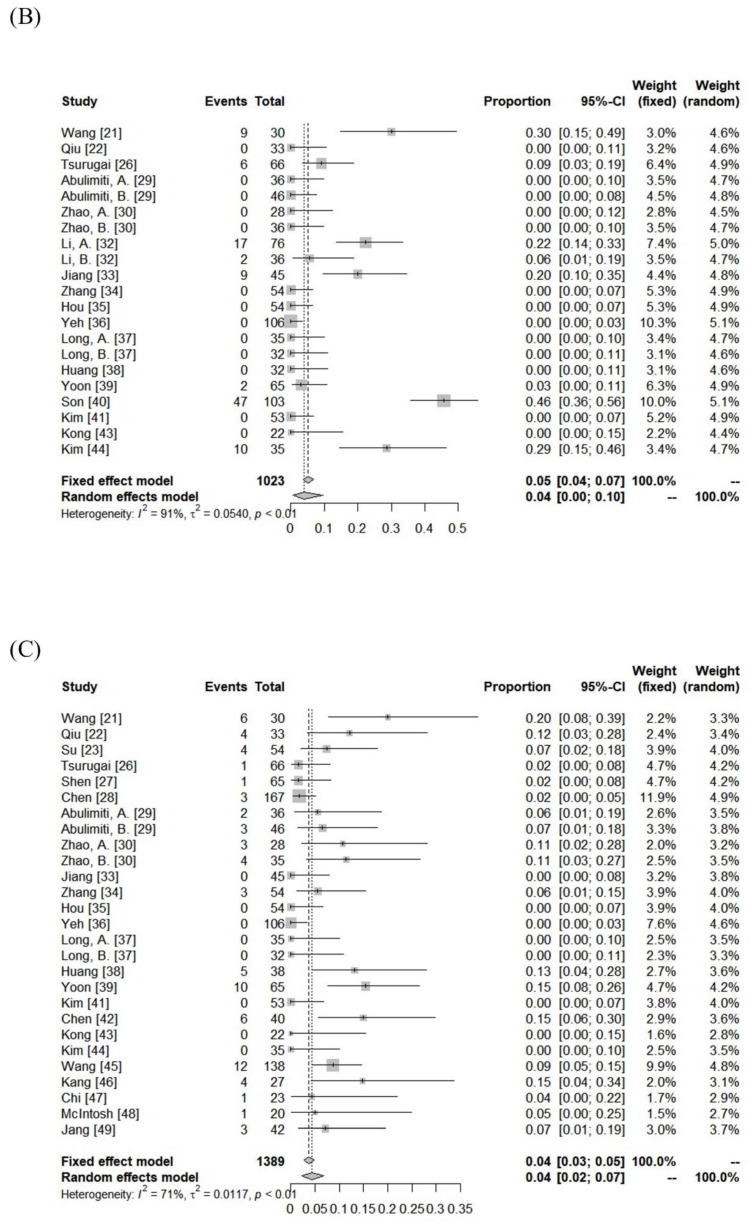

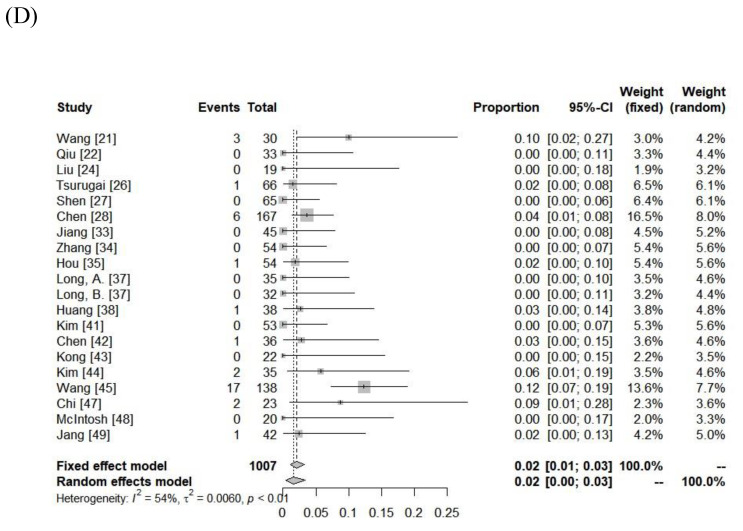
Forest plot of (**A**) classic radiation-induced liver disease (RILD), (**B**) nonclassic RILD, (**C**) acute hepatic toxicity ≥ grade 3, and (**D**) gastrointestinal toxicity ≥ grade 3.

The incidence of RILD was variable among the included cohorts (classic RILD = 0–31%; nonclassic RILD = 0–46%). The pooled rates assessed using the random-effects model for classic RILD and nonclassic RILD were 2% (95% CI, 0–6%) and 4% (95% CI, 0–10%), respectively. Severe hepatic toxicity and GI toxicity ≥ grade 3 were 6% (range: 0–20%) and 1% (range: 0–12%). The pooled rates of hepatic toxicity and GI toxicity ≥ grade 3 were 4% (95% CI, 2–7%) and 2% (95% CI, 0–3%), respectively. The toxicity data are summarized in Figure 3 and Supplementary Table S2.

## 4. Discussion

To our knowledge, this is the first systematic review and meta-analysis to focus on the treatment outcomes of IMRT for liver HCC. The theoretical advantages of IMRT dose distributions over two-dimensional RT and 3DCRT are generally accepted, and IMRT has become routinely used for both curative and palliative RT throughout all tumor sites [50]. Although IMRT has changed the practice of RT, it is unclear whether the use of IMRT can obtain a clinically relevant advantage over non-modulated RT. Three systematic reviews were conducted to investigate the evidence supporting the routine use of IMRT at various disease sites [8,9,51]. Toxicity reduction has been corroborated in head and neck cancer and breast cancer IMRT by several randomized trials, favoring IMRT for other tumor sites. However, the benefits of IMRT for LC and survival remain inconclusive. No randomized or prospective studies have compared IMRT and 3DCRT in the treatment of HCC. Existing data on 3DCRT for HCC show a wide variation in treatment outcomes according to the stage: 1-year LC and OS rates are 71–94% and 58–81% for early-stage, and 28–61% and 36–84% for advanced-stage HCC [4,52]. Our systematic review and meta-analysis reported that the LC and OS rates after IMRT were 84% (95% CI, 70–94%) and 59% (95% CI, 52–66%) at 1 year and 64% (95% CI, 45–81%) and 23% (95% CI, 14–33%) at 3 years, respectively. Considering that the included studies involved an advanced stage (median proportion of BCLC C stage: 100%) and a large tumor size (median: 7.4 cm), the current evidence of survival with IMRT for HCC is similar to that of 3DCRT, which is similar to other cancers. To improve the efficacy of IMRT, appropriate clinical studies using advanced technologies, such as image-guided RT, gating RT, altered fractionation, and adaptive RT, are needed.

Since the early 1960s, the use of EBRT for HCC has been limited because the entire liver can be treated safely only with 30–35 Gy in conventional fractionation. In the 1990s, however, the introduction of 3DCRT allowed the delivery of a high dose to the focal liver and quantification of the potential risk of RILD [53]. RILD is classified into classic RILD and nonclassic RILD, and RILD, mainly classic RILD occurs in 0–20% after 3DCRT [54,55]. In contrast, nonclassic RILD mainly occurs after SBRT but its incidence is low. Considering the low incidence of RILD after SBRT, variations in the definition of RILD among studies, and the difference in dose distribution between 3DCRT and SBRT being related to different repair mechanisms, recent studies have additionally reported hepatic toxicity, based on CTCAE, to sustain consistent reporting and compare other treatment modalities [54,56]. A meta-analysis of EBRT for HCC reported that the pooled rates of hepatic toxicity ≥ grade 3 were 10% (95% CI, 6–16%) for 3DCRT and 5% (95% CI, 3–8%) for SBRT [10]. Another HCC SBRT meta-analysis showed a pooled rate of hepatic toxicity ≥ grade 3 of 4.7% (95% CI, 3–7%) [57]. Recent SBRT meta-analysis focusing on small HCC ≤6 cm reported that the pooled rates of hepatic toxicity ≥ grade 3 and RILD were 4% (95% CI, 0–16%) and 15% (95% CI, 7–25%) [58]. Our meta-analysis showed that the pooled rates of classic RILD, nonclassic RILD, and hepatic toxicity ≥ grade 3 after IMRT were 2% (95% CI, 0–6%), 4% (95% CI, 0–10%), and 4% (95% CI, 2–7%), respectively. Although most studies had MVI (median proportion: 100%) and two-thirds of the included studies were treated with combined modalities in this meta-analysis, the current evidence of hepatic toxicity after IMRT for HCC is lower than that after 3DCRT and comparable with that after SBRT. Considering the slightly different toxicity criteria used in this study, further clinical studies would be needed to strengthen the safety of IMRT for HCC.

Another important issue regarding RT-related toxicity in patients with HCC is the risk of GI toxicity. Because the majority of HC patients have underlying liver cirrhosis or portal hypertension, they have a higher risk of GI ulcers, as well as portal hypertensive congestive gastropathy [59]. The incidence of GI toxicity in HCC patients who were treated with 3DCRT and underwent endoscopy was 30–50%, and V_25Gy_ or V_35Gy_ of the gastroduodenum (GD) were significant dosimetric parameters affecting severe GI toxicities [60,61,62]. Several dosimetric studies comparing 3DCRT and IMRT have shown that IMRT can spare high-dose regions of the GD, as mentioned above, and would reduce the risk of GI toxicity [16]. The simultaneously integrated boost (SIB) technique, a unique IMRT technology that applies different doses to different targets simultaneously, is another method used to reduce GI toxicity when the tumor is located near the GI organ. Five studies of this meta-analysis applied the SIB technique [24,27,37,41,49], and only one study [49] reported a grade 3 GI toxicity rate of 2%.

With respect to SBRT, one phase 2 study for HCC reported severe GI toxicity ≥ grade 3 of 11% [63]. The authors conducted a subsequent analysis of patients who underwent SBRT for abdominopelvic malignancies and found that the maximal point dose of the GD and a history of ulcer before SBRT were the best predictors of GI toxicity [64]. A further phase 2 SBRT study for HCC using different constraints according to endoscopic findings before SBRT showed severe GI toxicity ≥ grade 3 of 2% [65]. Another possible risk factor of GI toxicity after SBRT is the targeted agent [66,67]. Sorafenib, a multi-tyrosine kinase inhibitor, has been recommend for BCLC stage C since 2007, and Princess Margaret Hospital conducted a phase 1 study of SBRT combined with sorafenib (400 mg/day) [68,69]. Among HCC patients with a high veff of 30–60%, two of the three evaluable patients experienced grade 3–4 GI toxicity. The authors decreased the sorafenib tolerance dose to 200 mg and suggested that concurrent SBRT with sorafenib is not recommended outside clinical trials. On the other hand, a phase 2 study using IMRT combined with sorafenib reported a grade 3 GI toxicity of 3%, although the median tumor size was 8 cm (range, 3–16 cm) [42]. Another possible agent is bevacizumab, which an anti-vascular endothelial growth factor. Meta-analysis for bevacizumab reported that patients treated with bevacizumab had a significantly increased risk of gastrointestinal perforation compared with patients treated with control medication, with a relative risk of 2.14 (95% CI 1.19–3.85; *p* = 0.011) [70]. Barney et al. [71] reported a 35% risk of severe GI toxicity ≥ grade 3 who received bevacizumab after SBRT for intra-abdominal lesions, including HCC. One strategy to minimize GI toxicity after RT in patients treated with bevacizumab is to increase fractionation compared to SBRT [66]. The BCLC 2022 guidelines recommend a combination therapy of immunotherapy and bevacizumab as the first regimen for BCLC stages B and C [72]. Our meta-analysis including the combined treatment with a targeted agent showed GI toxicity ≥ grade 3 of 2% (95% CI, 0–3%), respectively, and we suggest that IMRT using multifraction is considerable treatment option for patients with a tumor located close to or attached to a GI organ, or patients with large-sized HCC treated with a targeted agent.

There are some limitations in this meta-analysis. First, there was no randomized trial, and either prospective or retrospective studies were included. The meta-analysis of observational studies is controversial [73]. Uncontrolled confounders may have affected the pooled analysis. Second, IMRT has the possibility of an increased risk of secondary cancer than 3DCRT [74]. Until now, there were no clinical data about secondary cancer after RT for HCC. When IMRT is considered as the best treatment option to ablate the tumor and decrease toxicity, the benefit of tumor control and safety outweighs the risk of possible long-term consequences [75]. Further clinical studies should be needed to find the optimal delivery method of IMRT and minimize the potential risk of secondary cancer. Lastly, IMRT is a kind of advanced RT technique and is sometimes used in SBRT. In addition, the definition of SBRT with a small number of fractions is somewhat ambiguous, and various fractionation schemes have been used for HCC. No definite criteria exist for the classification of fractionated SBRT and IMRT. Therefore, we artificially categorized RT using ≥10 fractions as IMRT, considering that the hypofractionation of ≥10 fractions is generally accepted to be biologically different from SBRT. The widespread routine use of IMRT extends beyond its rational use for all tumor sites, and IMRT is now the de facto standard [9]. Therefore, there is a need to accumulate evidence to justify IMRT, and our meta-analysis focusing on IMRT for HCC is meaningful in a real clinical setting.

## 5. Conclusions

IMRT is used worldwide at all tumor sites for both curing and palliation. While evidence exists for lower normal tissue toxicity, IMRT did not show a definitive survival benefit compared to 3DCRT. This is the first systematic review and meta-analysis to evaluate the rationale for using IMRT for HCC of the liver. Although most patients had advanced-stage HCC and combined treatment was commonly used, IMRT for HCC showed similar survival rates to existing RT modalities and relatively low severe toxicity. Further clinical trials are needed to improve treatment efficacy and to select patients who can benefit from fractionated IMRT.

## Figures and Tables

**Table 1:** Study details for hepatocellular carcinoma treated with intensity-modulated radiotherapy.

Author	Year	Country	Study Type	NOS	N	CP Class A/B/C (%)	BCLC Stage 0/A/B/C/D (%)	Median Size (cm) (Range)	MVI (%)	DM (%)	AFP ≥400 ng/mL (%)	Median Total Dose (Gy) (Range)	Dose per Fraction (Gy)	Combined Treatment
Wang [21]	2023	China	P	5	30	77/23/0	0/0/0/100/0	NR	100	53	60	NR (52–56)	2	Atezolizumab + bevacizumab (100%)
Qiu [22]	2023	China	P	4	33	76/18/6	0/3/55/42/0	8 (0.7–20.3)	NR	21	36	NR	2–3.5	Apatinib (100%)
Su [23]	2022	China	R	6	54	74/26/0	0/0/6/94/0	NR	NR	20	46	48	3	PD-1 inhibitors + anti-angiogenic (100%)
Liu [24]	2022	China	P	5	19	53/47/0	0/5/26/69/0	NR	47	26	32	62 (45–72)/45 (30–50) ^b^	3–4.5/2–2.5 ^b^	Apatinib (100%)
Li [25]	2022	China	R	4	109	NR	0/30/13/57/0	5.8 ^a^ ± 3.5	NR	NR	NR	50 (40–66)	1.8–4	No
Tsurugai [26]	2021	Japan	R	4	66	92/8/0	32/23/6/38/1	2.3 ^a^ (1.5–3.3)	NR	NR	NR	42	3	No
Shen [27]	2021	China	P	5	65	78/22/0	0/15/42/43/0	NR	NR	40	NR	50/30 ^b^	5/3 ^b^	Sorafenib (20%); Cytokines (63%)
Chen [28]	2021	China	R	6	167	93/7/0	0/0/0/100/0	NR	100	0	51	58 (50–67)	2–2.2	TACE (39%); Sorafenib (11%)
Abulimiti, A. [29]	2021	China	R	7	36	92/3/0	0/0/0/100/0	NR	100	0	53	NR (40–62.5)	2–2.5	Sorafenib (100%)
Abulimiti, B. [29]	2021	China	R	7	46	96/4/0	0/0/0/100/0	NR	100	0	48	NR (40–62.5)	2–2.5	No
Zhao, A. [30]	2019	China	R	6	28	100/0/0	0/0/0/100/0	7.4 (1.9–14.9)	100	0	43	NR	NR	TACE + sorafenib (100%)
Zhao, B. [30]	2019	China	R	7	35	100/0/0	0/0/0/100/0	6.6 (1.2–17)	100	0	43	NR	NR	TACE (100%)
Lo [31]	2019	Taiwan	R	6	23	39/52/9	0/0/0/96/4	NR	100	0	NR	NR (45–70)	3–4.5	TACE (30%)
Li, A. [32]	2018	China	R	6	76	96/4/0	0/0/0/100/0	NR	100	0	57	58 (50–67)	2–2.2	TACE (100%)
Li, B. [32]	2018	China	R	6	36	89/11/0	0/0/0/100/0	NR	100	0	67	58 (50–67)	2–2.2	TACE (100%)
Jiang [33]	2017	China	R	7	45	89/11/0	NR	NR	0	0	24	54 (35–68)	2.2–5.5	No
Zhang [34]	2016	China	R	5	54	100/0/0	NR	6.3 (1.2–14)	48	0	NR	50 (44–70)	1.8–2	TACE (100%)
Hou [35]	2016	China	R	8	54	85/15/0	0/0/0/100/0	7.5 ^a^ ± 3.5	100	0	NR	60 (40–66)	2.5–4	No
Yeh [36]	2015	Taiwan	R	5	106	78/22/0	0/0/0/100/0	NR	100	0	NR	60 (NR)	2	No
Long, A. [37]	2015	China	P	5	35	NR	NR	NR (10–20)	NR	0	NR	NR (40–50/30–40) ^b^	2 ^b^	No
Long, B. [37]	2015	China	P	5	32	NR	NR	NR (10–20)	NR	0	NR	NR (40–50/30–40) ^b^	2 ^b^	No
Huang [38]	2015	Taiwan	R	5	38	71/29/0	NR	4.6 (2.5–16.7)	NR	0	40	54 (46–71.8)	1.8–2.4	No
Yoon [39]	2014	Republic of Korea	R	8	65	100/0/0	NR	9.0 (2.2–18.8)	NR	0	NR	50 (47.5–60)	2.5–3.5	IA_CTx (95%)
Son [40]	2014	Republic of Korea	R	5	103	88/12/0	NR	6.2 ^a^ ± 6.6	NR	0	35	50 (40–60)	1.8–5	No
Kim [41]	2014	Republic of Korea	R	5	53	94/6/0	NR	5.5 (1.6–16)	66	0	NR	55 (55–66)/44 (44–55) ^b^	2.5–3/2–2.5 ^b^	Sorafenib (9.4%)
Chen [42]	2014	Taiwan	P	6	40	100/0/0	0/10/25/65/0	8.2 (3–15.5)	NR	0	NR	50 (40–60)	2–2.5	Sorafenib (100%)
Kong [43]	2013	Republic of Korea	R	5	22	68/32/0	NR	4.4 (0.9–16.4)	NR	0	NR	NR (30–60)	1.8–4	No
Kim [44]	2013	Republic of Korea	R	5	35	80/20/0	0/0/0/100/0	NR	100	0	NR	50 (45–60)	4.5–6	Capecitabine (100%)
Wang [45]	2012	Taiwan	R	5	138	70/30/0	0/6/21/73/0	10 (3.8–19.1)	NR	16	43	60 (45–66)	1.8–2	No
Kang [46]	2011	Republic of Korea	R	4	27	70/30/0	NR	11.4 (8.1–18.2)	67	0	NR	50.4 (45–64.8)	1.8	TACE or IA_CTx (48%)
Chi [47]	2010	Taiwan	R	5	23	65/35/0	NR	NR	74	22	NR	52.5 (NR)	2.5–4.5	Sunitinib (100%)
McIntosh [48]	2009	USA	R	5	20	55/45/0	NR	9 ^a^ (1.3–17.4)	NR	0	NR	50 (50–60)	2.5	Capecitabine (100%)
Jang [49]	2009	Republic of Korea	R	5	42	76/22/2	NR	9 ^a^ ± 3.2	NR	100	NR	51.03 (30–57.61) ^b^	3–5.7 ^b^	Capecitabine (100%)

NOS—the Newcastle–Ottawa Scale; N—number; CP—Child–Pugh; BCLC—Barcelona Clinic Liver Cancer; MVI—macrovascular invasion; DM—distant metastases (not including regional lymph node involvement); AFP—Alpha-fetoprotein; P—prospective; R—retrospective; NR—not reported; PD-1—programmed cell death protein 1; TACE—transarterial chemo-embolization; IA_CTx—intra-arterial chemotherapy. ^a^ Means’ mean value. ^b^ Applied simultaneous integrated boost technique.

**Table 2:** Treatment outcomes for hepatocellular carcinoma treated with intensity-modulated radiotherapy.

Author	Median f/u (mo)	Response Criteria	CR/PR/SD/PD (%)	ORR (%)	DCR (%)	1-year LCR (%)	Median OS (mo)	1-year OS (%)	2-year OS (%)	3-year OS (%)
Wang [21]	7.4	mRECIST	13/77/7/3	90	97	NR	9.8	48	NR	NR
Qiu [22]	11.4	RECIST	3/12/67/18	15	82	NR	NR	66.2	60	60
Su [23]	12	mRECIST	2/41/48/9	43	91	NR	20.1	72	28	NR
Liu [24]	9	mRECIST	31/37/21/11	68	89	NR	not reached	54.6	NR	NR
Li [25]	20.5	NR	NR	NR	NR	NR	25.7	77	56	NR
Tsurugai [26]	24	mRECIST	NR	NR	NR	92	NR	90	75	60
Shen [27]	24	NR	28/69/3 ^a^	97	97	100	18	75.4	43	36.2
Chen [28]	NR	NR	NR	NR	NR	NR	16.5	66.8	29.9	10.6
Abulimiti, A. [29]	11	RECIST	0/61/28/11	61	89	NR	11	44.8	3.8	NR
Abulimiti, B. [29]	9	RECIST	2/44/28/26	46	74	NR	9	28.6	2.6	NR
Zhao, A. [30]	13	mRECIST	11/36/28/25	47	75	NR	19	72	48	36
Zhao, B. [30]	14.1	mRECIST	0/46/31/23	46	77	NR	15.2	78	16	0
Lo [31]	NR	NR	NR	NR	NR	NR	8	NR	NR	NR
Li, A. [32]	11	mRECIST	13/46/33/8	59	92	NR	9.6	38.2	18.4	9.2
Li, B. [32]	14	mRECIST	19/53/22/6	72	94	NR	13.4	61.1	27.8	16.7
Jiang [33]	51.9	RECIST	9/49/38/4	58	96	NR	44.7	93.3	73.3	50
Zhang [34]	28.7	mRECIST	20/54/15/11	74	89	84.3	20.2	84.6	49.7	36.7
Hou [35]	11.8	RECIST	6/65/9/20	71	80	NR	15.47	59.3	32.1	26.4
Yeh [36]	10	WHO	10/52/33/5	62	95	NR	7	34.7	11	NR
Long, A. [37]	NR	mRECIST	6/29/48/17	35	83	NR	9.7	48.6	17.1	2.9
Long, B. [37]	NR	mRECIST	3/22/56/19	25	81	NR	6.5	21.9	0	0
Huang [38]	17.2	RECIST	5/48/34/13	53	87	88.2	12.6	56.2	31.7	NR
Yoon [39]	21	NR	NR	NR	NR	72.1	20	74	47.1	33.4
Son [40]	11.6	NR	NR	NR	NR	NR	11.6	48.5	23.4	14.3
Kim [41]	18.9	mRECIST	22/40/34/4	62	96	NR	25.1	NR	54.7	NR
Chen [42]	23	RECIST	2/53/37/8	55	92	44.6	14	56.7	32	NR
Kong [43]	14.4	mRECIST	18/55/23/4	73	96	NR	14.4	86.4	69.1	69.1
Kim [44]	12.9	RECIST	14/29/51/6	43	94	NR	12.9	51.4	22.2	9.2
Wang [45]	9	RECIST	11/53/29/7	64	93	NR	10.3	45	28	NR
Kang [46]	5	RECIST	4/41/33/22	45	78	NR	5	22	11	7.3
Chi [47]	16	RECIST	9/65/22/4	74	96	82	16	70	NR	NR
McIntosh [48]	NR	RECIST	NR	66	88	NR	9.6	40	30.6	NR
Jang [49]	9.4	RECIST	0/45/48/7	45	93	79	12.3	50.1	14.9	NR

f/u—follow-up; CR—complete response; PR—partial response; SD—stable disease; PD—progressive disease; ORR–objective response rate (CR + PR); DCR—disease control rate (CR + PR + SD); LCR—local control rate; OS—overall survival; mRECIST—modified Response Evaluation Criteria in Solid Tumors; NR—not reported. ^a^ 2 patients (3%) showed unmeasurable tumor.

**Table 3:** Pooled rates of treatment outcomes.

Group		Cohorts (N)	Patients (N)	P, Heterogeneity	I^2^	Egger’s Test, P	Fixed Event Rate (95% CI)	Random Event Rate (95% CI)	P (between Groups)
**All Patients**									
CR rate		26	1162	<0.0001	69.58%	0.4670	0.09 (0.07–0.11)	0.08 (0.05–0.12)	
ORR		26	1162	<0.0001	85.52%	0.2163	0.60 (0.57–0.62)	0.58 (0.50–0.65)	
DCR		26	1162	0.0014	51.31%	0.0488	0.91 (0.89–0.92)	0.90 (0.87–0.93)	
1-year LCR		8	393	<0.0001	90.31%	0.3638	0.85 (0.82–0.89)	0.84 (0.70–0.94)	
2-year LCR		8	423	<0.0001	92.94%	0.2007	0.77 (0.73–0.81)	0.75 (0.58–0.89)	
3-year LCR		3	185	0.0009	85.64%	0.9537	0.64 (0.57–0.71)	0.64 (0.45–0.81)	
1-year PFS		17	887	<0.0001	84.96%	0.7418	0.34 (0.31–0.37)	0.34 (0.25–0.42)	
2-year PFS		16	798	<0.0001	78.78%	0.3311	0.11 (0.09–0.14)	0.10 (0.06–0.16)	
3-year PFS		6	394	<0.0001	87.57%	0.1326	0.06 (0.04–0.09)	0.09 (0.02–0.20)	
1-year OS		31	1679	<0.0001	88.29%	0.8894	0.60 (0.57–0.62)	0.59 (0.52–0.66)	
2-year OS		29	1660	<0.0001	91.25%	0.8428	0.31 (0.29–0.34)	0.31 (0.23–0.39)	
3-year OS		18	978	<0.0001	92.11%	0.5075	0.22 (0.19–0.24)	0.23 (0.14–0.33)	
Subgroup									
1-year OS	mSize ≤7 cm	7	427	<0.0001	88.14%	0.5353	0.73 (0.69–0.77)	0.75 (0.61–0.86)	0.0180
	mSize >7 cm	9	447	<0.0001	77.16%	0.8765	0.54 (0.50–0.59)	0.55 (0.44–0.65)	
	mRT dose <51 Gy	11	638	<0.0001	88.45%	0.2042	0.68 (0.65–0.72)	0.65 (0.53–0.76)	0.3345
	mRT dose ≥51 Gy	11	744	<0.0001	87.94%	0.5229	0.55 (0.51–0.59)	0.57 (0.46–0.68)	
	Without TA/IT	21	1184	<0.0001	91.30%	0.8436	0.57 (0.55–0.60)	0.58 (0.48–0.67)	0.3144
	With TA/IT	10	495	0.0369	49.59%	0.2449	0.65 (0.61–0.69)	0.64 (0.57–0.70)	
2-year OS	mSize ≤7 cm	8	480	< 0.0001	90.74%	0.9223	0.46 (0.42–0.51)	0.47 (0.32–0.62)	0.1360
	mSize >7 cm	9	447	<0.0001	76.84%	0.8425	0.32 (0.28–0.37)	0.33 (0.23–0.43)	
	mRT dose <51 Gy	11	638	<0.0001	88.20%	0.2813	0.41 (0.37–0.45)	0.38 (0.27–0.50)	0.3426
	mRT dose ≥51 Gy	10	755	<0.0001	89.11%	0.3927	0.28 (0.25–0.32)	0.31 (0.21–0.42)	
	Without TA/IT	21	1184	<0.0001	92.48%	0.6977	0.30 (0.28–0.33)	0.29 (0.20–0.39)	0.4302
	With TA/IT	8	476	<0.0001	86.32%	0.7224	0.34 (0.30–0.39)	0.35 (0.24–0.48)	
3-year OS	mSize ≤7 cm	5	280	<0.0001	95.50%	0.7577	0.29 (0.23–0.34)	0.31 (0.08–0.60)	0.9724
	mSize >7 cm	5	207	0.0002	81.93%	0.9202	0.32 (0.26–0.39)	0.32 (0.17–0.48)	
	mRT dose <51 Gy	7	415	<0.0001	90.19%	0.6635	0.29 (0.25–0.33)	0.27 (0.14–0.42)	0.5148
	mRT dose ≥51 Gy	5	378	<0.0001	89.05%	0.2229	0.17 (0.13–0.21)	0.21 (0.09–0.35)	
	Without TA/IT	14	685	<0.0001	92.30%	0.6434	0.21 (0.18–0.24)	0.20 (0.10–0.32)	0.2961
	With TA/IT	4	293	<0.0001	93.55%	0.0970	0.22 (0.18–0.28)	0.34 (0.13–0.59)	

N—number; CI—confidence interval; CR—complete response; ORR—objective response rate (CR + partial response); DCR—disease control rate (CR + partial response + stable disease); LCR—local control rate; PFS—progression-free survival; OS—overall survival; mSize—median size; mRT dose—median radiotherapy dose; TA—target agent; IT—immunotherapy.

## Data Availability

All data generated or analyzed during this study are included in this published article.

## Supplementary Materials

The following supporting information can be downloaded at: https://www.mdpi.com/article/10.3390/cancers15204914/s1, Figure S1: Publication biases of included studies using Egger’s test and the funnel plots; Table S1: Search strategy and results; Table S2: Treatment toxicities.
