# Supplementary material for: The Current Evidence of Intensity-Modulated Radiotherapy for Hepatocellular Carcinoma: A Systematic Review and Meta-Analysis
Source: Cancers (Basel). 2023 Oct 10;15(20):4914. doi: 10.3390/cancers15204914 (PMC10605127; doi:10.3390/cancers15204914)
Supplement: Supplementary file 1 [file cancers-15-04914-s001.zip › cancers-2597322-supplementary/20231004 cancers-2597322-supplementary Figure S1.pdf]

Supplementary Figure S1. Publication biases of included studies using Egger's test and the funnel plots. (A) Objective response rate; (B) Disease control rate; (C) 1-year local control; (D) 3-year local control; (E) 1-year progression-free survival; (F) 3-year progression-free survival; (G) 1-year overall survival; (H) 3-year overall survival.

(A)

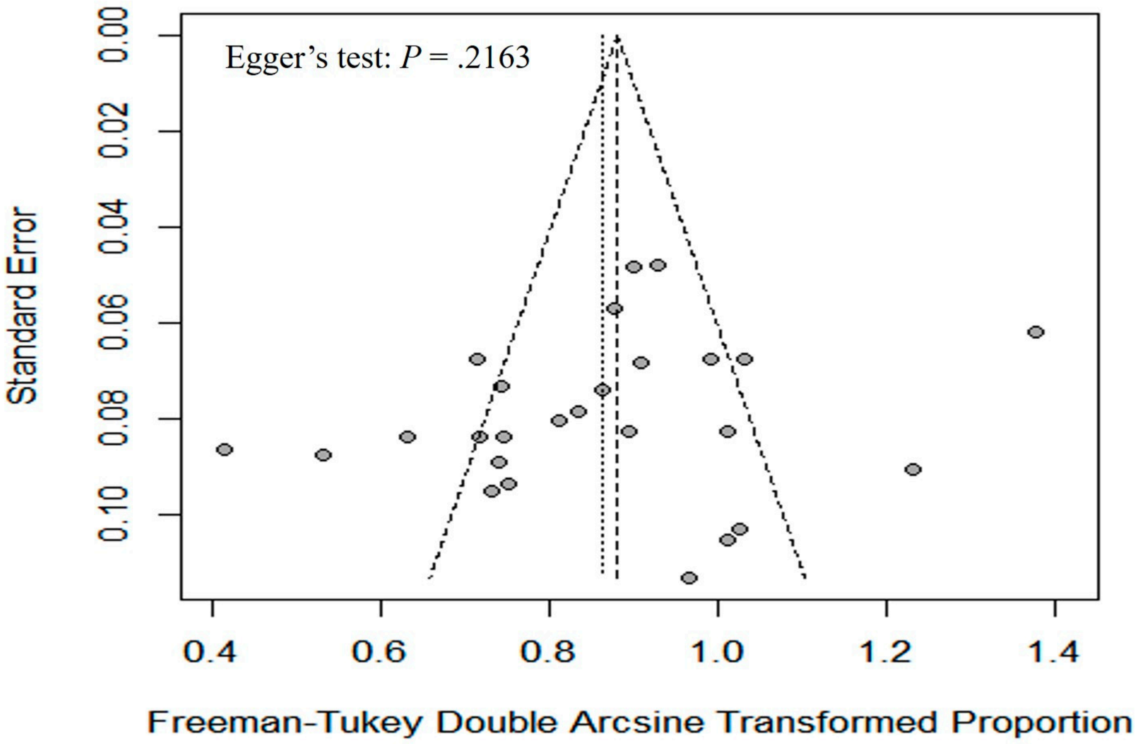

(B)

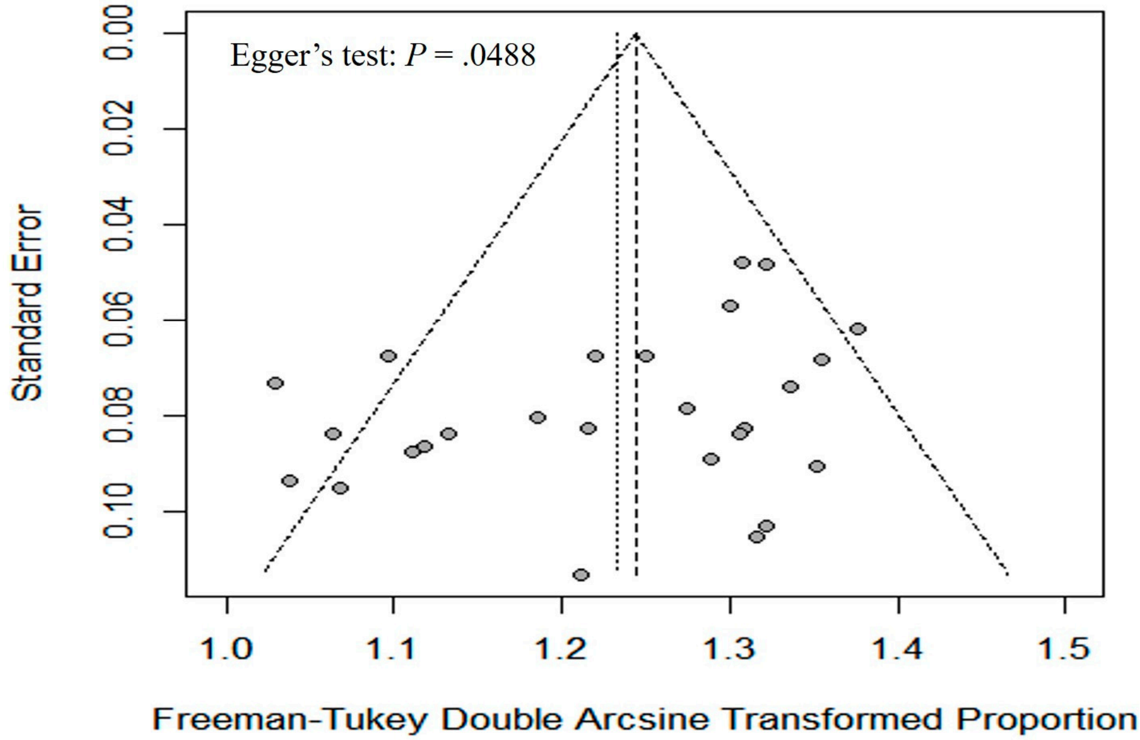
Egger's test: *P* = .0488
Standard Error
0.00
0.02
0.04
0.06
0.08
0.10
1.0
1.1
1.2
1.3
1.4
1.5
Freeman-Tukey Double Arcsine Transformed Proportion

(C)

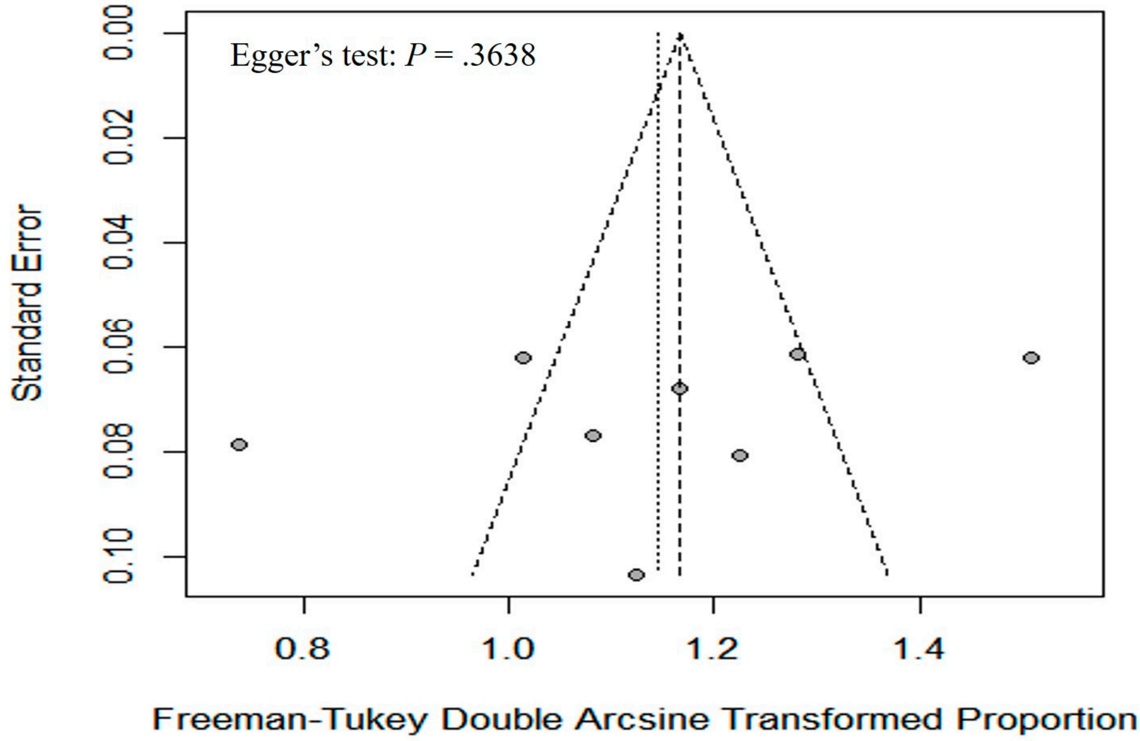
Egger's test: *P* = .3638
Standard Error
0.00
0.02
0.04
0.06
0.08
0.10
0.8
1.0
1.2
1.4
Freeman-Tukey Double Arcsine Transformed Proportion

(D)

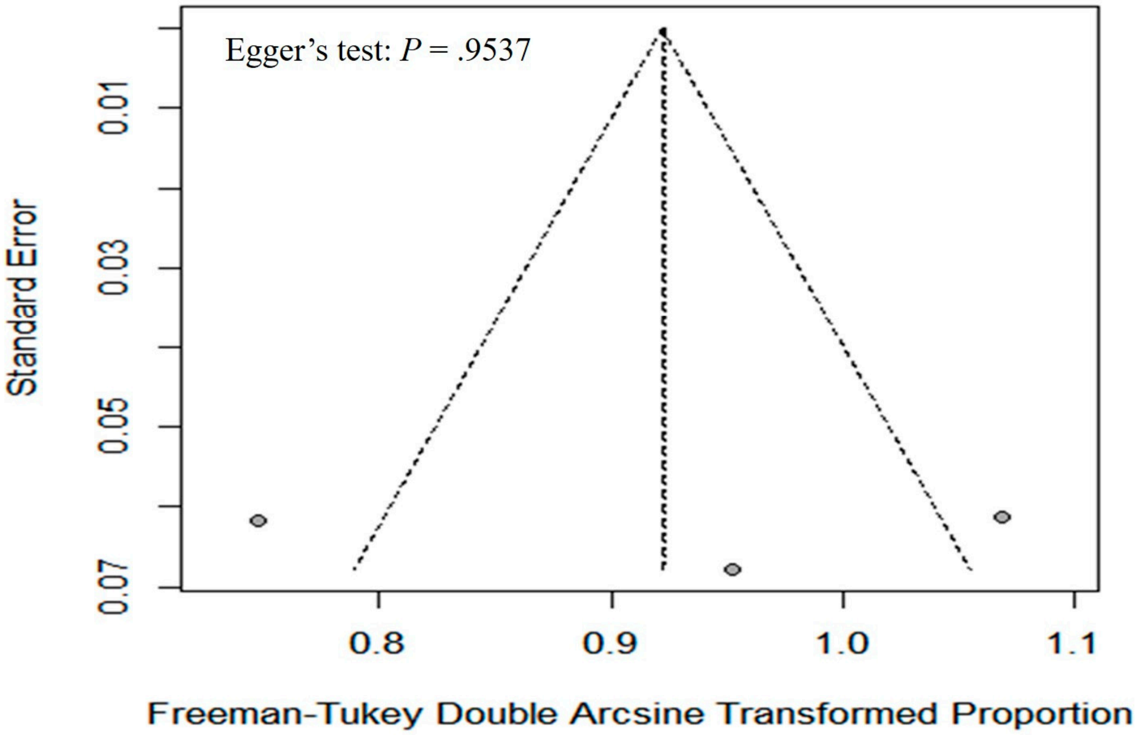

Egger's test: *P* = .9537
Standard Error
0.01
0.03
0.05
0.07
0.8
0.9
1.0
1.1
Freeman-Tukey Double Arcsine Transformed Proportion

(E)

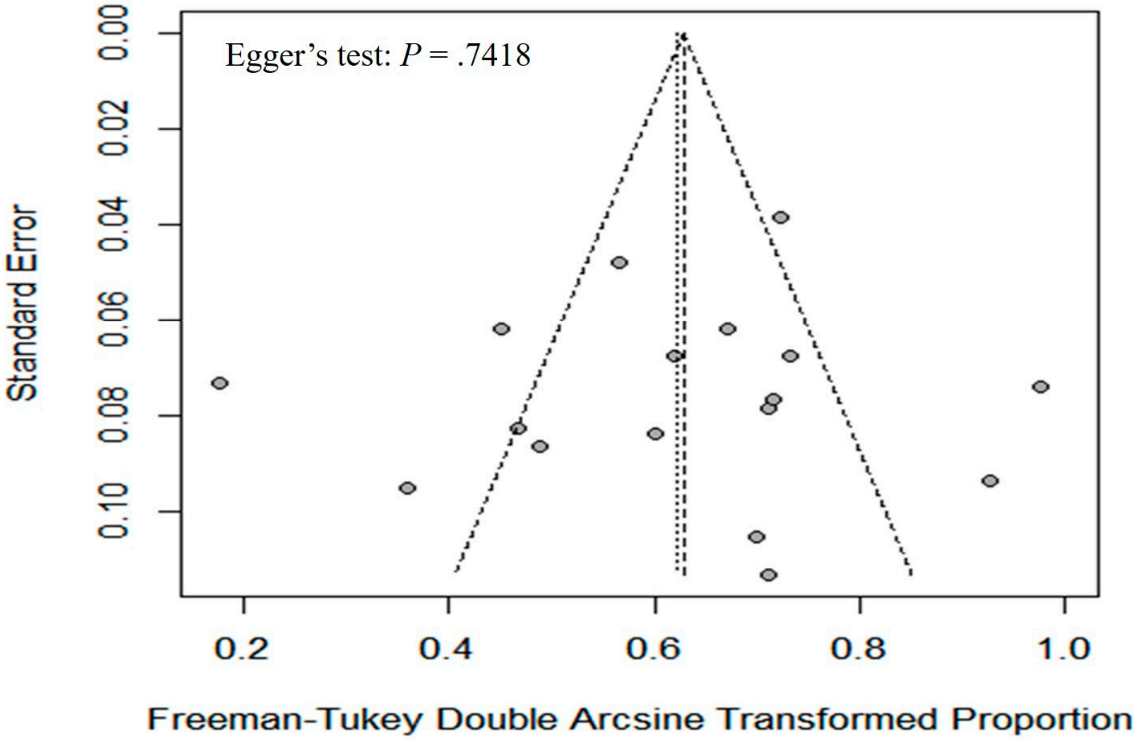

Egger's test: *P* = .7418
Standard Error
0.00
0.02
0.04
0.06
0.08
0.10
0.2
0.4
0.6
0.8
1.0
Freeman-Tukey Double Arcsine Transformed Proportion

(F)

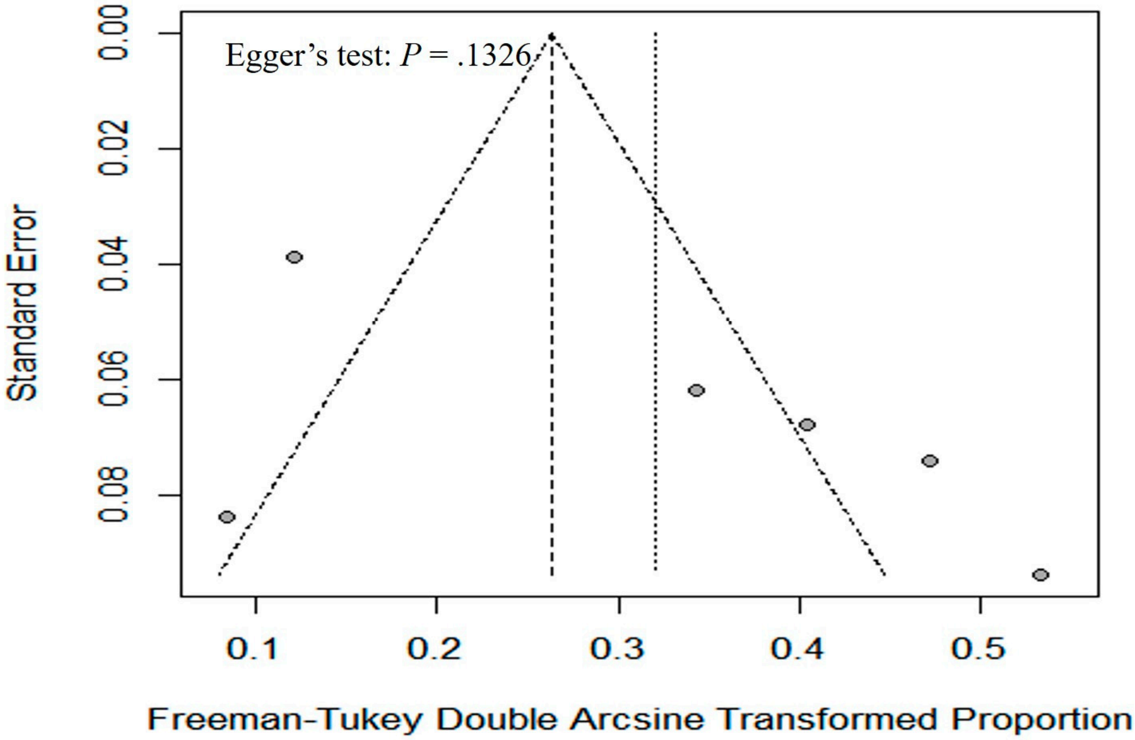
Egger's test: P = .1326
Standard Error
0.00
0.02
0.04
0.06
0.08
0.1
0.2
0.3
0.4
0.5
Freeman-Tukey Double Arcsine Transformed Proportion

(G)

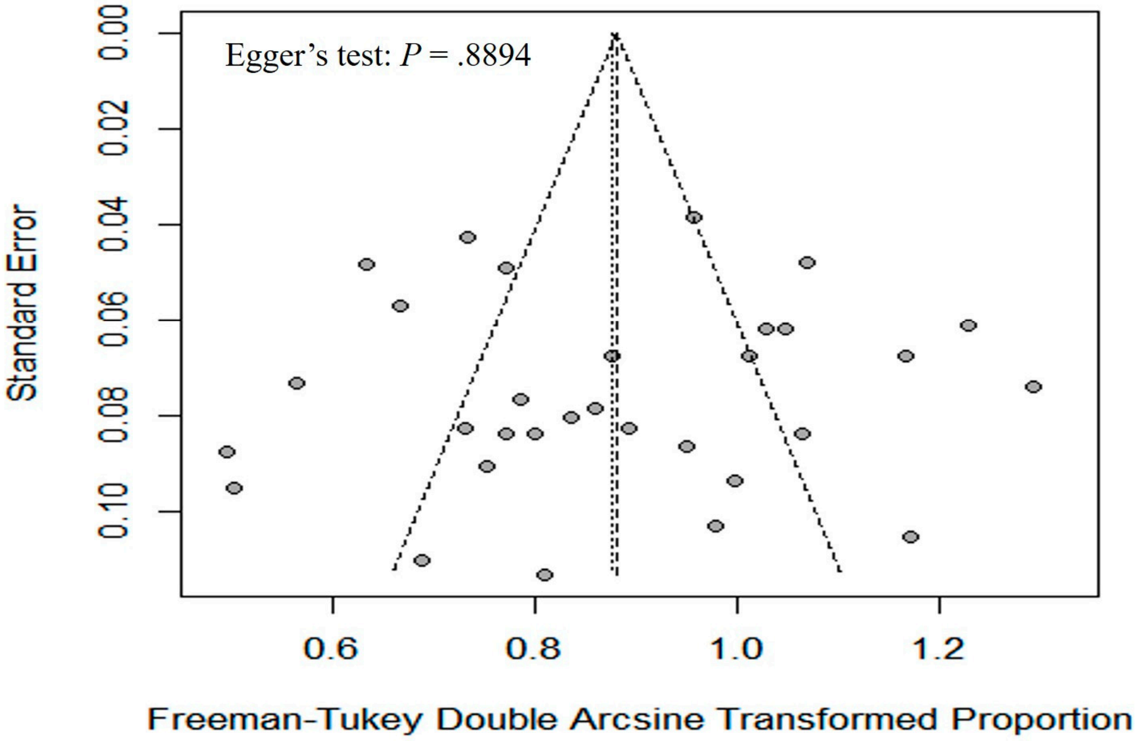
Egger's test: P = .8894
Standard Error
0.00
0.02
0.04
0.06
0.08
0.10
0.6
0.8
1.0
1.2
Freeman-Tukey Double Arcsine Transformed Proportion

(H)

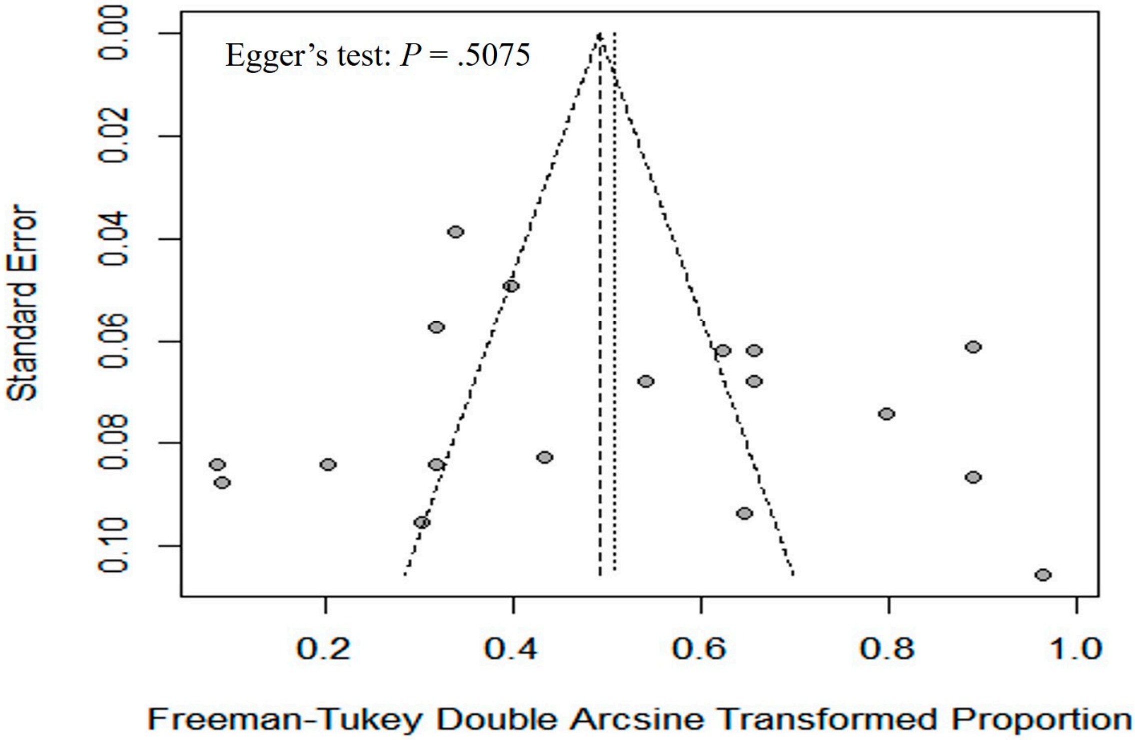
Egger's test: P = .5075
Standard Error
0.00
0.02
0.04
0.06
0.08
0.10
0.2
0.4
0.6
0.8
1.0
Freeman-Tukey Double Arcsine Transformed Proportion
