# Supplementary material for: The Current Evidence of Intensity-Modulated Radiotherapy for Hepatocellular Carcinoma: A Systematic Review and Meta-Analysis
Source: Cancers (Basel). 2023 Oct 10;15(20):4914. doi: 10.3390/cancers15204914 (PMC10605127; doi:10.3390/cancers15204914)
Supplement: Supplementary file 1 [file cancers-15-04914-s001.zip › cancers-2597322-supplementary/20231004 cancers-2597322-supplementary_tables_revised.pdf]

Table S1. Search strategy and results.

(1) Keywords

PICO	Fields	Keywords	Remarks
P	MeSH	Carcinoma, hepatocellular<br>Liver neoplasms	A
	Emtree	liver cell carcinoma<br>liver tumor<br>liver cancer	
	TIAB	Hepatocellular<br>Liver<br>Hepati*	B
		Neoplasm*<br>Tumor*<br>Tumour*<br>Carcinoma*<br>Cancer*<br>Malignan*	C
		**B AND C**	D
		Hepatoma*<br>HCC	E
		**A OR D OR E**	F
I	MeSH	Radiotherapy, intensity-modulated	G
	Emtree	intensity modulated radiation therapy<br>volumetric modulated arc therapy	
	TIAB	Intensity-modulated<br>Volumetric-modulated<br>Helical tomotherap*<br>Targeted radiation<br>IMRT	H

		**G OR H**	I
O	MeSH	Survival<br>Progression-free survival<br>Disease-free survival	J
	Emtree	survival<br>progression free survival<br>disease free survival<br>event free survival<br>overall survival	
	TIAB	Surviv*<br>Progression free surviv*<br>Event-free surviv*<br>Disease free surviv*<br>Overall surviv*	K
		**J OR K**	L
		**F AND I AND L**	

(2) Search strategy

DB	Search Strategy
**PubMed**	("carcinoma, hepatocellular"[MeSH Terms] OR "liver neoplasms"[MeSH Terms] OR (("Hepatocellular"[Title/Abstract] OR "Liver"[Title/Abstract] OR "hepati*"[Title/Abstract]) AND ("neoplasm*"[Title/Abstract] OR "tumor*"[Title/Abstract] OR "tumour*"[Title/Abstract] OR "carcinoma*"[Title/Abstract] OR "cancer*"[Title/Abstract] OR "malignan*"[Title/Abstract])) OR ("hepatoma*"[Title/Abstract] OR "HCC"[Title/Abstract])) AND ("radiotherapy, intensity modulated"[MeSH Terms] OR ("Intensity-modulated"[Title/Abstract] OR "Volumetric-modulated"[Title/Abstract] OR "helical tomotherap*"[Title/Abstract] OR "targeted radiation"[Title/Abstract] OR "IMRT"[Title/Abstract])) AND ("survival"[MeSH Terms] OR "progression free survival"[MeSH Terms] OR "disease free survival"[MeSH Terms] OR ("surviv*"[Title/Abstract] OR "progression free surviv*"[Title/Abstract] OR "event free surviv*"[Title/Abstract] OR "disease free surviv*"[Title/Abstract] OR "overall surviv*"[Title/Abstract]))

**EMBASE**	(('liver cell carcinoma'/exp OR 'liver tumor'/exp OR 'liver cancer'/exp) OR (('hepatocellular':ti,ab OR 'liver':ti,ab OR 'hepati*':ti,ab) AND ('neoplasm*:ti,ab OR tumor*:ti,ab OR tumour*:ti,ab OR carcinoma*:ti,ab OR cancer*:ti,ab OR malignan*:ti,ab)) OR (hepatoma*:ti,ab OR hcc:ti,ab)) AND (('intensity modulated radiation therapy'/exp OR 'volumetric modulated arc therapy'/exp) OR ('Intensity-modulated':ti,ab OR 'Volumetric-modulated':ti,ab OR 'Helical tomotherap*':ti,ab OR 'Targeted radiation':ti,ab OR 'IMRT':ti,ab)) AND (('survival'/exp OR 'progression free survival'/exp OR 'disease free survival'/exp OR 'event free survival'/exp OR 'overall survival'/exp) OR ('surviv*':ti,ab OR 'progression free surviv*':ti,ab OR 'event-free surviv*':ti,ab OR 'disease free surviv*':ti,ab OR 'overall surviv*':ti,ab))
**Cochrane Library**	(((MeSH descriptor: [Carcinoma, Hepatocellular] explode all trees OR MeSH descriptor: [Liver Neoplasms] explode all trees)) OR (((Hepatocellular):ti,ab,kw OR (Liver):ti,ab,kw OR (Hepati*):ti,ab,kw) AND ((Neoplasm*):ti,ab,kw OR (Tumor*):ti,ab,kw OR (Tumour*):ti,ab,kw OR (Carcinoma*):ti,ab,kw OR (Cancer*):ti,ab,kw OR (Malignan*):ti,ab,kw)) OR ((Hepatoma*):ti,ab,kw OR (HCC):ti,ab,kw)) AND ((MeSH descriptor: [Radiotherapy, Intensity-Modulated] explode all trees) OR ((Intensity-modulated):ti,ab,kw OR (Volumetric-modulated):ti,ab,kw OR (Helical tomotherap*):ti,ab,kw OR (Targeted radiation):ti,ab,kw OR (IMRT):ti,ab,kw)) AND ((MeSH descriptor: [Survival] explode all trees OR MeSH descriptor: [Progression-Free Survival] explode all trees OR MeSH descriptor: [Disease-Free Survival] explode all trees) OR ((Surviv*):ti,ab,kw OR (Progression free surviv*):ti,ab,kw OR (Event-free surviv*):ti,ab,kw OR (Disease free surviv*):ti,ab,kw OR (Overall surviv*):ti,ab,kw)) NOT (MeSH descriptor: [Animals] this term only NOT (MeSH descriptor: [Animals] this term only AND MeSH descriptor: [Humans] this term only))
**Web of Science**	(((((((TI=(Hepatocellular)) OR TI=(Liver)) OR TI=(Hepati*)) OR AB=(Hepatocellular)) OR AB=(Liver)) OR AB=(Hepati*)) AND (((((((((((TI=(Neoplasm*)) OR TI=(Tumor*)) OR TI=(Tumour*)) OR TI=(Carcinoma*)) OR TI=(Cancer*)) OR TI=(Malignan*)) OR AB=(Neoplasm*)) OR AB=(Tumor*)) OR AB=(Tumour*)) OR AB=(Carcinoma*)) OR AB=(Cancer*)) OR AB=(Malignan*)) OR ((((TI=(Hepatoma*)) OR TI=(HCC)) OR AB=(Hepatoma*)) OR AB=(HCC))) AND ((((((((((TI=("Intensity-modulated")) OR TI=("Volumetric-modulated")) OR TI=("Helical tomotherap*")) OR TI=("Targeted radiation")) OR TI=("IMRT")) OR AB=("Intensity-modulated")) OR AB=("Volumetric-modulated")) OR AB=("Helical tomotherap*")) OR AB=("Targeted radiation")) OR AB=("IMRT")) AND ((((((((((TI=(Surviv*)) OR TI=("Progression free surviv*")) OR TI=("Event-free surviv*")) OR TI=("Disease free surviv*")) OR TI=("Overall surviv*")) OR AB=("Surviv*")) OR AB=("Progression free surviv*")) OR AB=("Event-free surviv*")) OR AB=("Disease free surviv*")) OR AB=("Overall surviv*"))

**KoreaMed**	((((("Carcinoma, hepatocellular"[MH])) OR ("Liver neoplasms"[MH])) OR (((((("Hepatocellular"[TIAB])) OR ("Liver"[TIAB])) OR ("Hepatic"[TIAB])) OR ("Hepatitis"[TIAB])) AND (((((((("Neoplasm"[TIAB])) OR ("Tumor"[TIAB])) OR ("Tumour"[TIAB])) OR ("Carcinoma"[TIAB])) OR ("Cancer"[TIAB])) OR ("Malignant"[TIAB])) OR ("Malignanacy"[TIAB]))) OR ((("Hepatoma"[TIAB])) OR ("HCC"[TIAB]))) AND ((("Radiotherapy, intensity-modulated"[MH])) OR ((((((("Intensity-modulated"[TIAB])) OR ("Volumetric-modulated"[TIAB])) OR ("Helical tomotherapy"[TIAB])) OR ("Helical tomotherapies"[TIAB])) OR ("Targeted radiation"[TIAB])) OR ("IMRT"[TIAB]))) AND ((((("Survival"[MH])) OR ("Progression-free survival"[MH])) OR ("Disease-free survival"[MH])) OR (((((((("Survival"[TIAB])) OR ("Survive"[TIAB])) OR ("Progression free survival"[TIAB])) OR ("Event-free survival"[TIAB])) OR ("Disease free survival"[TIAB])) OR ("Overall survival"[TIAB])) OR ("Overall survive"[TIAB])))) NOT (("Animals"[MH]) NOT (("Animals"[MH]) AND ("Humans"[MH])))

(3) Summary of search results

No	Databases	Results	Duplication
1	PubMed	212	384
2	EMBASE	500	
3	Cochrane Library	62	
4	Web of Science	171	
5	KoreaMed	4	
Number of Search results (with duplication)		949	
**Number of Search results (without duplication)**		**565**	

Table S2. Treatment toxicities.

Author	Classic RILD (%)	Nonclassic RILD (%)	Acute hepatic toxicity ≥ grade 3 (%)	GI ulcer/bleeding/perforation ≥ grade 3 (%)
Wang [21]	0	30	20	10
Qiu [22]	0	0	12.1	0
Su [23]	NR	NR	7.4	NR
Liu [24]	NR	NR	NR	0
Li [25]	NR	NR	NR	NR
Tsurugai [26]	NR	9.1	1.5	1.5
Shen [27]	NR	NR	1.5	0
Chen [28]	NR	NR	1.8	3.6
Abulimiti, A. [29]	0	0	5.6	NR
Abulimiti, B. [29]	0	0	6.5	NR
Zhao, A. [30]	0	0	10.7	NR
Zhao, B. [30]	0	0	11.4	NR
Lo [31]	NR	NR	NR	NR
Li, A. [32]	NR	22.4	NR	NR
Li, B. [32]	NR	11.1	NR	NR
Jiang [33]	0	20	0	0
Zhang [34]	0	0	5.6	0
Hou [35]	0	0	0	1.9
Yeh [36]	2.8	0	0	NR
Long, A. [37]	11.4	0	0	0
Long, B. [37]	31.3	0	0	0
Huang [38]	2.6	0	13.2	2.6
Yoon [39]	0	3.1	15.3	NR
Son [40]	NR	45.6	NR	NR
Kim [41]	0	0	0	0
Chen [42]	15	NR	15	2.8
Kong [43]	4.6	0	0	0
Kim [44]	0	28.6	0	5.7

Wang [45]	24.6	NR	8.7	12.3
Kang [46]	NR	NR	14.8	NR
Chi [47]	NR	NR	4.3	8.7
McIntosh [48]	NR	NR	5	0
Jang [49]	NR	NR	7.1	2.4

Pooled rates of toxicities

Group	Cohorts (n)	Patients (n)	*P*, Heterogeneity	I^2	Egger's test, *P*	Fixed Event rate (95% CI)	Random Event rate (95% CI)
Classic RILD	19	925	< .0001	85.20%	0.1222	0.03 (0.02-0.05)	0.02 (0.00-0.06)
Nonclassic RILD	21	1023	< .0001	91.31%	0.1742	0.05 (0.04-0.07)	0.04 (0.00-0.10)
Acute HT ≥ grade 3	27	1389	< .0001	70.54%	0.1694	0.04 (0.03-0.05)	0.04 (0.02-0.07)
GI toxicity ≥ grade 3	20	1007	0.0021	54.11%	0.1315	0.02 (0.01-0.03)	0.02 (0.00-0.03)

RILD–radiation induced liver disease; GI–gastrointestinal; NR–not reported; HT–hepatic toxicity; CI–confidence interval.
